# Measles and Rubella Diagnostic and Classification Challenges in Near- and Post-Elimination Countries

**DOI:** 10.3390/vaccines12060697

**Published:** 2024-06-20

**Authors:** Thomas D. Filardo, Stephen N. Crooke, Bettina Bankamp, Kelley Raines, Adria D. Mathis, Tatiana M. Lanzieri, R. Suzanne Beard, Ludmila Perelygina, David E. Sugerman, Paul A. Rota

**Affiliations:** Division of Viral Diseases, National Center for Immunization and Respiratory Diseases, Centers for Disease Control and Prevention, Atlanta, GA 30333, USA; qjf9@cdc.gov (S.N.C.); bfb9@cdc.gov (B.B.); pvw2@cdc.gov (K.R.); xda5@cdc.gov (A.D.M.); uyk4@cdc.gov (T.M.L.); zho3@cdc.gov (R.S.B.); ifw0@cdc.gov (L.P.); ggi4@cdc.gov (D.E.S.); par1@cdc.gov (P.A.R.)

**Keywords:** measles, rubella, elimination, viral diagnostics

## Abstract

Measles and rubella are vaccine-preventable viral diseases and can be prevented by safe, highly effective vaccination with measles- and rubella-containing vaccines. Given the myriad causes of febrile exanthems, laboratory surveillance for both measles and rubella is important to document the incidence of these diseases and to track the progress and maintenance of elimination in near- and post-elimination settings. Diagnostic challenges can hinder effective surveillance and classification challenges can hinder efforts to demonstrate achievement or maintenance of elimination. In this report, we review diagnostic and classification challenges for measles and rubella in near- and post-elimination settings.

## 1. Introduction

Measles and rubella are vaccine-preventable viral diseases. Both can be prevented by safe, highly effective vaccination with measles- and rubella-containing vaccines, including measles–mumps–rubella (MMR) and measles–rubella (MR) formulations. The elimination of measles and rubella indicates the absence of a continuous endemic transmission of these viruses within a country. In 2012, the World Health Assembly endorsed the Global Vaccine Action Plan (GVAP), with the goal of achieving measles elimination in at least five World Health Organization (WHO) regions by 2020 [1,2]. However, by the end of 2020, none of the six WHO regions had achieved and sustained elimination. The GVAP also included a target to achieve the elimination of rubella in at least five of the six WHO regions by 2020; the Region of the Americas has maintained the elimination of rubella and congenital rubella syndrome (CRS) since 2015 [3]. The WHO remains committed to achieving and sustaining measles and rubella elimination globally, per the Immunization Agenda 2030 [4].

Given the myriad causes of febrile exanthem in both elimination and endemic settings, laboratory surveillance for measles and rubella is important to document the incidence and accurately describe the epidemiology of these diseases so that immunity gaps can be efficiently targeted. Robust laboratory surveillance is key to documenting the course towards elimination in settings where measles and rubella circulate endemically; laboratory surveillance is also critical for documenting the maintenance of elimination. The WHO Global Measles and Rubella Laboratory Network (GMRLN) provides laboratory support for measles and rubella surveillance on a global scale. GMRLN laboratories perform laboratory testing for case confirmation and use molecular epidemiologic methods to monitor viral transmission pathways [5].

Diagnostic challenges can hinder effective surveillance efforts for measles and rubella, particularly accurate and effective contact investigations, which are key components of achieving and maintaining elimination. The classification of cases is also an integral part of achieving and demonstrating the ongoing elimination of measles and rubella at a national and subnational level. Case classification addressed in this discussion includes laboratory confirmation of a measles or rubella case (or the discarding of a suspected case); the differentiation of primary or secondary vaccine failure cases when measles or rubella occurs among those with pre-existing immunity; the determination of vaccine-associated reactions which can mimic measles or rubella; and the classification of measles or rubella cases by source of infection (i.e., imported, import-associated, endemic, or unknown) [6].

In this article, we use a series of fictional, but relatively common, patient-based scenarios to review ongoing diagnostic and classification challenges for measles and rubella which exist in near- and post-elimination countries. These scenarios draw on the experience of measles and rubella national surveillance teams at the United States Centers for Disease Control and Prevention (CDC); these six scenarios are drawn from multiple different suspected and confirmed measles and rubella cases and are fictionalized to maintain privacy.

## 2. Diagnostic Scenarios and Discussion of Management

### 2.1. Scenario 1

A 4-year-old child was seen by her primary care provider with a 2-day history of fever and maculopapular rash. Additional symptoms included coryza and a mild cough that preceded the rash by 3 days. There was no reported exposure to measles, and no reported domestic or international travel in the 21 days prior to symptom onset. The child had received a single dose of MMR vaccination according to the routine U.S. vaccination schedule at 12 months of age [7]. Laboratory testing was limited to the detection of measles-specific IgM in serum, which returned a positive result. Due to delayed reporting, the local public health agency was notified of the positive result 7 days after the case’s rash onset, at which time the child had fully recovered.

A public health investigation began. Given that there had been no reported measles cases during the prior 6 months in the state and county of the child’s residence, and there were no clear risk factors for measles infection in the child, the possibility of a false–positive measles IgM was considered.

#### 2.1.1. Diagnostic Strategies for Scenario 1

The detection of measles-specific IgM has historically been the primary mode of laboratory confirmation of measles and is recommended by the WHO as the primary test for measles case confirmation. However, the limitations of IgM testing can be seen in settings with low measles incidence. In endemic settings, where the pre-test probability of measles for a patient presenting with a febrile exanthem is high, the positive predictive value (PPV) of IgM tests would be much higher than in elimination settings, where the PPV would be lower due to the low prevalence of the condition within the tested population [8,9]. The concern from the public health agency regarding the possibility of a false–positive IgM result in this scenario is warranted, given the lack of clear measles risk factors and lack of known regional transmission. Measles IgM is known to cross-react with antibodies against other causes of common childhood exanthems, including parvovirus B-19 and human herpesvirus 6 (HHV6), among others [10,11,12]. False–positive results can also be obtained due to antibody interference (e.g., rheumatoid factor) or other factors [13]. There are also many other potential viral causes of a febrile exanthem that can present clinically as a measles-like illness, including adenovirus, enterovirus, Epstein–Barr virus, and cytomegalovirus, although antibody cross-reactivity has not been routinely demonstrated for these pathogens [14,15].

Options for additional diagnostic testing to discern the likelihood of measles in this case include molecular and serologic testing.

Molecular detection may be challenging in this case since public health notification occurred 7 days after rash onset; however, real-time reverse-transcription polymerase chain reaction (rRT-PCR) can detect measles in respiratory and urine specimens for 10 or more days after rash onset, although the sensitivity of rRT-PCR drops sharply in all specimens after day 7 following rash onset [16,17,18]. The collection of both a respiratory and urine specimen may improve the sensitivity of testing overall, but negative results for rRT-PCR in this instance would not rule out measles given the declining sensitivity of rRT-PCR testing after day 7. If the original serum specimen from days 0 to 3 after rash onset was available for testing, attempting to detect measles using rRT-PCR in serum may be a viable option for confirming measles, although the sensitivity of serum rRT-PCR may be variable [18,19,20]. Additionally, serum is not a routinely validated specimen for measles rRT-PCR and testing it may not be possible for diagnostic purposes.

Additional serologic testing may provide supportive information. Serum IgM is generally present for 6–8 weeks after measles infection; therefore, repeating serum IgM may be an option if the patient is able to provide an additional blood specimen [21]. If the initial test was performed with an Enzyme Immunoassay (EIA) using an indirect format, the use of an IgM capture EIA to retest the acute specimen, or the testing of the convalescent specimen, may be beneficial given the generally higher sensitivity and specificity of capture tests compared to indirect tests [22,23]. If acute and convalescent specimens are available for analysis, demonstration of seroconversion or a 4-fold rise in IgG titer using a plaque reduction neutralization test (or another quantitative IgG test validated for this use) can provide additional evidence of acute measles infection. However, it should be noted that the plaque reduction neutralization test is a highly specialized assay that is not available in most laboratories conducting routine public health surveillance.

#### 2.1.2. Scenario 1 Classification Challenges

Additional diagnostic testing, as outlined above, may provide insight into the likelihood of measles infection in this scenario. However, in the absence of additional information, the classification of this case may be challenging in near- or post-elimination settings and may depend upon local guidance. For example, the detection of measles-specific IgM in the absence of recent vaccination is considered laboratory confirmation of measles in the United States, Japan, and England, whereas Canada requires epidemiologic linkage to a PCR positive measles case or known travel to an area of measles activity to consider IgM confirmatory, and Australian guidelines include IgM as “suggestive” but not confirmatory laboratory evidence of infection (Table 1). Guidance available from Mexico suggests that additional testing such as paired samples or IgG avidity can be used to classify IgM positive cases [24]. Therefore, heterogeneity in guidance from elimination settings may alter case classification in a similar scenario depending on the country and on the availability of additional diagnostic testing.

Additional classification challenges also can occur if serology alone is available for diagnosis. Cases can be classified according to importation status, i.e., whether they were related to importation into a country after exposure during international travel, or if a case was related to transmission occurring within a country. Without genotyping, the determination of the source of infection for the case may remain unknown. If many cases are reported with an unknown import status, this raises concern of missed importations and missed endemic transmission, which can complicate the case of achieving or sustaining elimination for near- and post-elimination countries.

### 2.2. Scenario 2

A 28-year-old woman presents for routine obstetric care at 12 weeks of gestation; as part of routine screening, testing for immunity to rubella is requested. She lives in a country that has verified rubella elimination. The submitting provider orders testing for both IgG and IgM, both of which return positive results. Public health is notified of the positive IgM result. The patient reported prior vaccination against rubella, although she does not have documentation of this vaccination. The public health investigation reveals that the patient traveled internationally 1 month ago for 2 weeks to a region that has not declared rubella elimination; the patient denies any illness prior to, during, or after travel, and does not report any sick contacts during travel. WHO has not reported any recent rubella cases in the WHO region of travel during the preceding 3 months.

#### 2.2.1. Diagnostic Strategies for Scenario 2

The detection of rubella-specific IgM remains the standard for laboratory confirmation of rubella infection. However, like measles, rubella IgM tests can be prone to false positivity. Causes for false–positive rubella IgM include cross-reactivity, test interference, or prolonged IgM positivity after infection or vaccination [25,26]. The diagnosis of acute rubella during pregnancy is critical in identifying congenital rubella syndrome, which can occur regardless of the clinical severity of maternal infection [26]. The diagnosis of rubella is also complicated by the high proportion of cases that may be asymptomatic [27].

To avoid unnecessary public health investigations, it is important to note that screening for rubella immunity should include only rubella IgG and should not include rubella IgM unless there is concern for recent or acute rubella infection. Testing using IgM for people without the risk of recent or acute rubella infection would be expected to result only in false–positive results.

Additional diagnostic testing for this case would rely on serological methods. Molecular detection of rubella could be attempted in this case, although the detection window for rubella using rRT-PCR is relatively narrow; rubella RNA is reliably detected for at least 4 days after rash onset [27]. However, detection of rubella RNA can occur further out from rash onset in some instances; in one study, up to 50% of oropharyngeal specimens were positive using RT-PCR around day 8–9 after rash onset [28].

The most informative study in this particular situation would be to perform IgG avidity on the original specimen, or a new specimen if the original is unavailable. Given the high efficacy of vaccine protection against rubella illness, high avidity IgG would suggest prior vaccination or remote (three or more months) exposure to rubella and would be inconsistent with a recent infection. The detection of low avidity IgG would confirm recent exposure to wild-type rubella (or rubella vaccination, if applicable). Repeat IgM could also be considered in this case to evaluate the possibility of a false–positive IgM, including a capture IgM if available at a reference laboratory. Given that the patient would be in the convalescent phase of illness, a repeat serum sample for paired IgG titers may not be informative. Of note, in an instance where serologic testing occurs during later stages of pregnancy, high avidity IgG would not be able to differentiate infection earlier in pregnancy (more than three months prior) from prior vaccination or remote exposure to rubella.

#### 2.2.2. Scenario 2 Classification Challenges

Additional diagnostic testing may provide insight into the likelihood of rubella infection in this scenario. However, in the absence of additional laboratory information, the classification of this case may be challenging in near- or post-elimination settings and may depend upon local guidance. As outlined in Table 1, the use of IgM testing as laboratory confirmation varies among different post-elimination settings. As with Scenario 1, additional classification challenges are introduced if serology alone is used for the confirmation of diagnosis, including the concern about missed endemic spread in near- or post-elimination settings.

### 2.3. Scenario 3

A 6-year-old male is seen in the emergency department (ED) for a two-day history of fever and cough, followed by an onset of a maculopapular rash just prior to presentation. He arrived in the United States from a measles-endemic country 14 days before being seen in the ED. Ten days ago, he was seen in a refugee clinic for intake examination and, given that he had no available prior vaccine records, was given a dose of MMR vaccine. With concern for measles illness, the emergency clinicians contact local public health authorities, who recommend rRT-PCR testing; rRT-PCR performed on a throat swab returns positive 2 days later, by which time the patient’s symptoms have resolved. Local and state public health authorities contact the CDC to help determine if the patient’s symptoms were due to wild-type measles infection or a reaction to MMR vaccination.

#### 2.3.1. Diagnostic Strategies for Scenario 3

Patients who receive live attenuated measles-containing vaccine may go on to develop self-limited fever and rash approximately 7–10 days after vaccination, which can be clinically indistinguishable from wild-type measles infection in some cases [21,29]. The presence of respiratory symptoms cannot rule out vaccine reaction, as it may be due to mild respiratory symptoms related to the vaccine reaction itself, or due to concurrent respiratory symptoms related to other infections that commonly occur in children at the age they generally receive MMR vaccination.

Measles can be detected using rRT-PCR during and after wild-type measles infection, as well as through the detection of vaccine-strain measles virus after vaccination with any live attenuated measles-containing vaccine (including MR and MMR). The duration of vaccine-strain detection is unclear; prolonged detection of measles vaccine strain (>100 days after vaccination) has been reported, although such prolonged detection appears to be rare [30]. The differentiation of vaccine strain from wild-type measles is critical when a person has both recent vaccination (i.e., within 21 days of onset of fever and rash) and an epidemiologic risk for measles, as in this case.

Differentiating wild-type from vaccine-strain measles virus can be performed by two methods. First, genotyping can differentiate the two, as the wild-type virus of genotype A (from which all global vaccine strains are derived) no longer circulates in any country globally and would only be detected after vaccination. However, genotyping is time consuming, and results may be required sooner to guide rapid public health response. Delayed recognition of a vaccine reaction by clinicians and public health practitioners may result in an unnecessary public health investigation which can be costly to jurisdictions and burdensome to patients and providers. Another option when rapid results are required is a specialized measles virus genotype A rRT-PCR (MeVA), which can provide rapid differentiation between vaccine strain and wild-type measles virus in approximately 3 h [31]. MeVA is performed in parallel with a standard measles virus rRT-PCR (MeV); a positive result for MeVA in conjunction with a positive MeV result confirms the detection of vaccine-strain measles virus.

In general, in post-elimination settings, MeVA is only required when there is a known epidemiologic risk for wild-type measles. If a patient experiences a febrile rash within 21 days of receipt of MMR (or other measles-containing vaccine), but there is no identified epidemiologic risk, then the syndrome could be clinically diagnosed as a vaccine reaction without additional testing. However, in near-elimination settings, or endemic settings, where epidemiologic risk may be more commonly encountered in domestic settings, further testing may be warranted in a wider set of circumstances.

#### 2.3.2. Scenario 3 Classification Challenges

In near-elimination and endemic settings, classifying vaccine reactions may require advanced molecular diagnostics. In this case, with both recent vaccination and epidemiologic risk for wild-type measles, serology and molecular diagnostics cannot differentiate the immune response to vaccination or the presence or absence of wild-type measles, respectively, without further testing. In post-elimination settings, epidemiologic risk can generally be more carefully defined (when there is no local active transmission), and classification can be performed without advanced diagnostics in most cases [32].

### 2.4. Scenario 4

A 24-year-old female presented for the evaluation of symptoms. She reported an onset of fever, cough, and coryza 6 days ago, and noticed a faint rash that appeared on her face 4 days ago; the rash subsequently generalized to her trunk and extremities. She was seen in the emergency department and held overnight for observation but released with improving symptoms 3 days ago; measles testing was not performed. On day 4 after rash onset, the patient’s primary care provider alerted the health department of the concern for measles. Vaccine records showed receipt of one dose of MMR at age 2 years. A serum and a nasopharyngeal swab were obtained the next day (5 days after rash onset); these specimens’ results returned IgG positive, IgM positive, and negative using rRT-PCR. The health department inquires if the case can be ruled out using negative rRT-PCR.

#### 2.4.1. Diagnostic Strategies for Scenario 4

The detection of measles virus using rRT-PCR generally has higher sensitivity and specificity than the detection of IgM, but there are important caveats to the use of rRT-PCR as a broad “rule-out” for measles. While sensitivity of rRT-PCR is high during days 0–3 following rash onset, some reports suggest that sensitivity drops after this timeframe [16,17]. Additionally, patients who develop measles despite pre-existing immunity (i.e., secondary vaccine failure cases) may have lower viral titers overall and may shed measles virus for a shorter time period, as evidenced by a lower risk of secondary transmission from these cases [33]. rRT-PCR sensitivity can also be negatively impacted if specimens are collected, stored, or handled improperly. Therefore, since the timeline for the collection of the specimen after rash onset is critical for the sensitivity and specificity of the rRT-PCR assay, the rRT-PCR results should always be interpreted in the context of epidemiologic risk, clinical presentation, and the results of other laboratory testing.

Additional serologic testing, if available, may provide clarity to the diagnosis of measles. The use of paired acute and convalescent serum specimens may confirm measles infection (as discussed in Scenario 1). Additionally, given that this case was IgG positive, testing for IgG avidity may be useful; the detection of low avidity IgG would confirm recent measles infection in the absence of recent vaccination [34].

#### 2.4.2. Scenario 4 Classification Challenges

As outlined, additional serologic testing may be helpful to provide diagnostic clarity for this case. If additional serologic testing is not available, either because specialized testing such as IgG avidity is unavailable or because a convalescent specimen cannot be obtained, prioritizing one diagnostic result over another may lead to classification challenges (i.e., using rRT-PCR to “rule out” measles cases despite positive serologic testing). Such broad use of rRT-PCR as a “rule out” test may result in missed cases if applied broadly.

### 2.5. Scenario 5

A 5-year-old male was seen in the emergency room with a 3-day history of subjective fever and coryza, followed by a generalized maculopapular rash that began on his chest and moved to his extremities. He had recently been exposed to a family member who had returned from a measles endemic country with febrile rash illness; however, measles testing had not been pursued for the family member. Due to mild symptoms, the patient’s family did not seek care until five days after rash onset. Serologic testing resulted in detectable anti-measles IgM and IgG, and measles virus RNA was detected using rRT-PCR from a nasopharyngeal swab. The patient had written documentation of one MMR dose at 12 months of age.

#### 2.5.1. Diagnostic Strategies for Scenario 5

In this case, the diagnostic challenge lies not in the confirmation of measles, but in classifying the cause of vaccine failure. It is important in elimination settings to attempt to classify measles cases which occur after vaccination as primary vaccine failure (PVF) or secondary vaccine failure (SVF), and to understand the role that vaccine failure cases play in measles outbreaks [35]. PVF to the measles component of MMR occurs in ~2–7% of recipients of MMR at 12 months of age. In PVF, there is a failure to seroconvert after receiving the measles vaccine [36]. In general, almost all people who fail to respond to a first dose would be expected to respond to a second dose with seroconversion, such that the VE for two-dose MMR recipients is higher (97%) than for one-dose recipients (93%) [37]. SVF rarely occurs, generally after prolonged or intense exposures in a household or other congregate setting. Vaccine-derived immunity is more variable and generally shorter lived than immunity acquired after measles infection, such that ~5% of children lose protective antibody levels 10–15 years after vaccination; however, protective immunity may be maintained even in those whose antibody levels decline over time [38]. Additionally, in near- and post-elimination settings, individuals are unlikely to encounter wild-type measles throughout their lifetime, such that there would not be a boosting effect to stimulate maintenance of immunity. Despite these challenges, measles remains largely a condition affecting those unvaccinated against it. During the 15 years after elimination was declared in the US (2001–2015), only 12.9% of measles cases occurred among people documented to have received measles vaccination [39].

#### 2.5.2. Scenario 5 Classification Challenges

The classification of vaccine failure cases relies largely on the measurement of IgG avidity. The presence of IgG at the time of rash onset or prior to rash onset is evidence of pre-existing immunity, and therefore is suggestive of SVF in vaccinated individuals (i.e., it suggests against primary vaccine failure and lack of prior seroconversion) [21]. However, measles is often not recognized until later in the disease course, and the presence of IgG later after rash onset can be characteristic of a primary or secondary immune response. Therefore, when anti-measles IgG is detected, the further characterization of IgG avidity can be used to classify vaccine failure. Low IgG avidity is suggestive of a primary immune response, and therefore suggestive of PVF, if prior vaccination has been documented [40]. High IgG avidity is characteristic of a mature immune response which develops over the course of months; therefore, detection of high avidity IgG is suggestive of SVF. Measles IgG avidity testing is available in specialized laboratories such as the U.S. CDC. Though measurement of IgG avidity is the primary method for discriminating between SVF and PVF, SVF can also be confirmed via detection of high titer (>40,000 mIU/mL) of measles-specific neutralizing antibodies; as previously mentioned, neutralization tests are only available in a few specialized laboratories [40].

### 2.6. Scenario 6

A jurisdiction in a country with sustained measles elimination reported an outbreak of 10 cases of measles because of a community spread of measles from an internationally imported case; transmission was documented in community settings including an elementary school and the waiting room of a healthcare facility. The outbreak was limited to a single county within that jurisdiction. Outbreak-associated cases had rash onsets which spanned from June to July of that year, and the outbreak was declared over after two incubation periods (i.e., 42 days) had passed after the last rash onset associated with the outbreak. Measles genotype D8 was detected in the outbreak.

In September of the same year, two months after the reporting of the outbreak-related cases, the jurisdiction is notified of a patient presenting with measles-compatible symptoms who subsequently tested positive for measles RNA using rRT-PCR; genotype D8 was detected. An epidemiologic investigation determined that the patient had no connection to the prior outbreak and had not traveled outside the country during their possible incubation period. Public health authorities sought to determine if the case is related to a separate importation event, or if measles may have resulted from a chain of undetected transmission related to the prior outbreak.

#### Classification Strategies for Scenario 6

In elimination settings, the identification of the source of infection for measles and rubella cases primarily relies on the epidemiologic investigation, and linkage to known cases or outbreaks can be performed based on epidemiologic investigation. However, such epidemiologic linkages cannot always be identified. Measles and rubella surveillance includes analysis of the molecular epidemiology of these viruses and is used to track importations and transmission patterns globally and within countries. Identical sequences from two cases can provide evidence of a shared chain of transmission, although it is important to note that without accompanying epidemiologic evidence, molecular epidemiology alone cannot prove a direct linkage between cases. Sequences from measles and rubella cases that are distinct can also provide evidence against linkage in the same chain of transmission if there is adequate resolution to identify the distinction between sequences.

Specimens from measles cases can be assigned to 1 of 24 genotypes based on the sequence of the 450 nucleotides coding for the carboxy (COOH) terminal 150 amino acids of the nucleoprotein gene (N-450) [41]. While WHO recognizes 24 genotypes, there has been a substantial reduction in the genetic diversity of circulating measles genotypes, and only genotypes B3 and D8 have been detected since 2021 [42]. Time-based phylogenetic analysis of sequence variations within a genotype can provide evidence for separate chains of transmission. However, the limited sequence information provided by N-450 may not always be sufficient to provide substantial evidence for or against local transmission. Sequencing additional regions of the measles genome by partial or whole-genome sequencing may be able to increase the resolution needed to distinguish between chains of transmission [43,44].

## 3. Discussion and Conclusions

Despite the availability of robust molecular and serologic laboratory tests for the confirmation of measles and rubella infection, diagnostic challenges for measles and rubella persist in near- and post-elimination settings. As highlighted in these scenarios, many diagnostic challenges can be addressed by the combined use of serology and molecular diagnostics, which can provide complementary information. Although rRT-PCR has high sensitivity and specificity during the optimal timeframe for collection (i.e., within 3 days after rash onset), serology maintains utility for cases where there is a delayed recognition of measles or rubella, in cases where presence of measles virus may be short-lived (e.g., in those with pre-existing immunity), and in documentation of prior immunity and classification of vaccine failure where appropriate. However, it is important to note that laboratory testing results alone cannot substitute for thorough epidemiologic investigation of all cases, since case confirmation and classification is based on the evaluation of laboratory and epidemiologic data.

We also highlight the additional diagnostic and classification challenges that can be addressed by advanced laboratory diagnostics, including the differentiation of vaccine strain from wild-type measles infection, and evaluation for undetected chains of transmission. The resolution of sequence analysis will improve as more laboratories move to whole-genome sequencing. For near-elimination countries, establishing a subnational or national network of laboratories capable of performing such advanced diagnostics is important to be able to demonstrate the achievement and maintenance of measles and rubella elimination. The availability of rapid diagnostic tests will also facilitate the development of subnational networks and will improve the time required to confirm measles cases [45]. The use of multiplex testing for a broader array of causes of febrile exanthems may also help detect more common viral etiologies of febrile rash in elimination settings that mimic measles and rubella and may cross-react with serologic testing [46].

The achievement and maintenance of measles and rubella elimination requires intensive case- and laboratory-based surveillance. Diagnostic and classification challenges persist in near- and post-elimination settings. Integrating epidemiologic and laboratory findings, including results from both serologic and molecular modalities, can help alleviate some diagnostic and classification challenges in these settings.

## Figures and Tables

**Table 1:** Use of IgM for measles and rubella case classification by the World Health Organization and select post-elimination countries.

Country/Jurisdiction	Detection of Measles- or Rubella-Specific IgM	Source
Australia	Laboratory suggestive evidence; can support probable case definition only.	Measles: https://www.health.gov.au/sites/default/files/documents/2022/06/measles-surveillance-case-definition.pdf Rubella: https://www.health.gov.au/sites/default/files/documents/2022/06/rubella-surveillance-case-definition.pdf
Canada	Laboratory confirmation only if case is epidemiologically linked to a PCR confirmed case or has recently travelled to an area of known measles activity.	Measles: https://www.bccdc.ca/health-professionals/clinical-resources/case-definitions/measles# Rubella: http://www.bccdc.ca/health-professionals/clinical-resources/case-definitions/rubella-(german-measles)#
England	Laboratory confirmation that can support confirmed case definition.	Measles: https://assets.publishing.service.gov.uk/media/65bb924dcc6fd600145dbe4d/20240123_national-measles-guidelines-February-2024.pdf Rubella: https://assets.publishing.service.gov.uk/media/5c35e849ed915d732cade0a5/UK_measles_and_rubella_elimination_strategy.pdf
Japan	Laboratory confirmation that can support confirmed case definition.	Measles: https://www.niid.go.jp/niid/images/iasr/35/410/de4101.pdf Rubella: https://www.niid.go.jp/niid/images/iasr/36/425/de4251.pdf
Mexico	Can support laboratory confirmation if additional serologic studies (e.g., avidity testing, paired sera) support acute measles infection.	Measles and Rubella: https://www.scielo.org.mx/scielo.php?pid=S0016-38132019000500492&script=sci_arttext&tlng=en
United States of America	Laboratory confirmation that can support confirmed case definition.	Measles: https://ndc.services.cdc.gov/case-definitions/measles-2013/ Rubella: https://ndc.services.cdc.gov/case-definitions/rubella-2013/
World Health Organization	Laboratory confirmation that can support confirmed case definition.	Measles: https://www.who.int/publications/m/item/vaccine-preventable-diseases-surveillance-standards-measles Rubella: https://www.who.int/publications/m/item/vaccine-preventable-diseases-surveillance-standards-rubella

Note: The access date for all listed websites was 15 March 2024.
